# Fabrication and Characterization of Taurine Functionalized Graphene Oxide with 5-Fluorouracil as Anticancer Drug Delivery Systems

**DOI:** 10.1186/s11671-021-03541-y

**Published:** 2021-05-13

**Authors:** Hao Pan, Yanjie Yu, Li Li, Bingmi Liu, Yu Liu

**Affiliations:** 1 School of Pharmacy, Liaoning University, Shenyang, 110036 China; 2 Liaoning Key Laboratory of New Drug Research & Development, Shenyang, 110036 China; 3 Liaoning Pharmaceutical Engineering Research Center for Natural Medicine, Shenyang, 110036 China

**Keywords:** Graphene oxide, Taurine, Nanomaterial, Toxicity, Bioavailability

## Abstract

Recently, nanocarrier systems for cancer drugs, especially GO-based drug delivery systems, have become a boon for cancer patients. In this study, we choose Tau to functionalize the GO surface to improve its biocompatibility. Firstly, nano-scale GO was synthesized by the modified Hummer’s method and ultrasonic stripping method. The taurine-modified graphene oxide carrier (Tau-GO) was synthesized by chemical method to obtain Tau-GO that has a good dispersibility and stability in water, with a zeta potential of − 38.8 mV and a particle size of 242 nm. Based on the encapsulation efficiency evaluation criteria, the optimal formulation was determined to combine Tau-GO and 5-FU by non-covalent bonding. The 5-FU-Tau-GO was more stable in neutral environment than in acidic environment, and with a certain PH response and sustained release effect. In vivo, we compared oral and intravenous administrations of 5-FU and 5-FU-Tau-GO, respectively, using pharmacokinetic tests and related parameters and showed that 5-FU-Tau-GO oral or intravenous administration prolongs the action time of 5-FU in the body and improves its bioavailability. In addition, the inhibition of HepG2 cells that was measured by the MTT assay, showed that the IC_50_ value of 5-FU was 196 ± 8.73 μg/mL, and the IC_50_ value of 5-FU-Tau-GO was 65.2 ± 0.7 μg/mL, indicating that 5- FU-Tau-GO is more potent against HepG2 cells and has a stronger inhibitory effect on cancer cells. The effect on cell morphology that was measured using the AO/EB staining also showed that 5-FU-Tau-GO not only disrupted cells, but also significantly induced apoptosis compared to 5-FU. We also verified by computer aided design that Tau-GO can bind better to 5-FU than to the unmodified GO, and that the formed 5-FU-Tau-GO system is more stable, and conducive to the transfer and release of 5-FU in vivo.

## Introduction

Chemotherapy is still a common method that is used in the treatment of various cancers [1]. A significant obstacle of most chemotherapeutic agents is their inability to penetrate tumor tissues, at effective concentrations, or their undesired side effects to normal tissues [2]. Therefore, scientists have concentrated their efforts on developing a potent drug delivery system that can achieve the controlled rate of drug release in tumor tissues to ensure effective drug delivery and therapy.

Many nanosized materials including liposomes [3], polymers [4], nanoparticles [5], dendrimers [6], micelles [7], and graphene oxide [8, 9] have been developed for the delivery of various drugs. Among these nanomaterials, graphene oxide (GO) is a novel carbon nanomaterial that is chemically exfoliated from oxidized graphite and exhibits several fascinating physical and chemical properties, such as abundant functional groups, large specific surface area, high drug-loading amount, and excellent dispersal ability in water [10–12]. Moreover, most of the in vitro experimental results showed that a low GO concentration could be used as substrate for cell growth and to activate immune cells. Therefore, GO has been widely used in disease diagnosis [13], cancer cell imaging and tracking [14], cancer photothermal therapy [15], tissue engineering [16], targeted drug delivery [17], and especially, as an anti-tumor drugs carrier [18, 19]. However, several research groups have reported that a high GO concentration has obvious cytotoxic effects in pre-clinical and clinical studies. The mechanism by which GO produces toxic effects in vivo is through oxidative stress and the overproduction of intracellular reactive oxygen species, inducting cell apoptosis and causing severe inflammation, pulmonary edema and granuloma formation [20]. Therefore, it is of critical importance to solve the issue of GO toxicity.

Reportedly, the functionalization of covalent or non-covalent bonds can reduce the strong hydrophobic interaction between GO and cells. This was demonstrated by several studies that showed that GO functionalization improves its biocompatibility and almost eliminate its toxicity in vivo and in vitro.Yang et al. studied, for the first time, the long-term biodistribution of covalently conjugating PEG-GO in mice using a radiolabeling method, and the results showed that PEG-GO could be gradually excreted from mice, likely in urine and feces [21]. Zhang et al. compared DEX functionalized GO with GO and found that GO-DEX could remarkably reduce cell toxicity and be mostly cleared from mice within a week [22]. In addition, GO non-covalent binding to pluronic F127 results in benign solubility and stability in physiological conditions, and a low toxicity [23]. Although several polymers or molecules have been used to functionalize GO, significant results have been achieved in this field. However, efforts are still needed to develop simple methods to construct a good biocompatible GO-based drugs’ carrier.

Taurine (Tau) is a semi-essential amino acid with good stability and water solubility. Tau can prevent cardiovascular and cerebrovascular diseases, maintain visual function, protect cells, and regulate immunity. Numerous studies have proposed that Tau has antitumor properties through its upregulation or downregulation of the expression factors that play important roles in various cancers, including lung, stomach, colorectal, and breast cancers [24]. Thus, Tau functionalized GO may exert a superior role as an anti-tumor drugs’ carrier. In this article, we used, for the first time, covalent Tau functionalized GO as a nanocarrier and evaluated its cytotoxicity in vitro. Furthermore, 5-fluorouracil (5-FU) was used as an anticancer drug that was non-covalently loaded on the surface of Tau-GO to build a drug delivery system. The 5-FU-Tau-GO could not only reduce the side effects on normal tissues but also improve drug bioavailability. Consequently, Tau-GO was successfully developed as a novel GO-based nanomaterial that may have significant biomedical applications in the future.

## Experiments and Methods

### Materials

The graphite powder, phosphate buffered saline (PBS) and dimethyl sulfoxide (DMSO) were purchased from Tianjin Laibo Chemical Co., Ltd.; Carbodiimide (EDC), N-hydroxysuccinimide (NHS), hydrochloric acid (HCl) and hydrogen peroxide (H_2_O_2_ 30%) were purchased from Shandong Yuwang Industrial Co., Ltd.; Taurine (Tau), 5-fluorouracil (5-FU), human hepatoma cells (HepG2), penicillin–streptomycin solution and fetal bovine serum (FBS) were purchased from Dalian Meilun Biotechnology Co., Ltd.; sulfuric acid (H_2_SO_4_ 98%) and potassium permanganate (KMnO_4_) were purchased from Nanjing Chemical Reagent Co., Ltd.; sodium nitrate (NaNO_3_) and sodium dodecyl sulfate (SDS-Na) were purchased from Shanghai Jinjinle Industrial Co., Ltd.; MTT was purchased from Sigma-Aldrich, Inc; DMEM was purchased from HyClone, Inc; The rats were purchased from the Benxi Changsheng Experimental Animal Center. All other reagents and chemicals were analytically pure and commercially available.

### GO Synthesis

The GO was prepared from graphite powder according to a modified Hummer’s method. Firstly, 168 mL of 98% sulfuric acid was added along the flask wall of a three-necked flask that was placed in an ice bath with thermometer, and 5 g graphite and 4 g sodium nitrate were added when the temperature reached 5 °C. Then, 22.5 g of potassium permanganate was slowly added in batches for 1 h and the temperature was kept below 5 °C. After that, the three-necked flask was transferred to an oil bath and the obtained mixture was reacted at 35 °C for 30 min, then the temperature was increased to 65 °C and the reaction stirred for 30 min. Following this step, the temperature was increased to 85 °C and the mixture was further reacted for 1 h to obtain a purple-brown paste. This mixture was left standing at room for 1 week, transferred to a beaker with 700 mL of hot water, and the 30% hydrogen peroxide was added dropwise until it became yellowish brown. The mixture was centrifuged at 10,000 rpm, washed with hot water, and this process was repeated several times until the pH of the supernatant was 7.0. Finally, the product was dried in a vacuum freeze dryer and the nano GO was obtained.

### Tau-GO Synthesis

An accurately weighed 50 mg GO was dissolved in 50 mL distilled water, and 150 mg EDC and 100 mg NHS were added to activate the GO by ultrasonication in an ice water bath for 20 min. Next, 10 mL Tau aqueous solution (0.1 g/mL) was slowly added (dropwise) into the prepared GO aqueous solution, and the pH was adjusted to 6–7 by HCl and continuously stirred for 24 h at room in the dark. The product was collected by centrifugation at 5000 rpm for 10 min and washed 3 times with distilled water. The Tau-GO was collected after freeze drying.

### 5-FU Loading

An amount of 20 ml Tau-GO solution was ultrasonicated for 2 h. An accurately weighed quantity of 5-FU was dissolved in an appropriate amount of distilled water, and slowly added dropwise to the prepared Tau-GO solution under stirring at room temperature, then sonicated at 30 °C in the dark for 1.5 h. The products were centrifuged at 5000 rpm for 10 min at 4 °C. The lower sediment was washed with 20 mL distilled water and centrifuged (5000 rpm for 10 min at 4 °C), and the process was repeated 3 times. The lower layer was freeze-dried, the supernatant was placed in a beaker, weighed the volume, the concentration of 5-FU was determined by a 1200 HPLC (Agilent, USA). The detection conditions were as follows: chromatographic column: C18 (4.6 × 250 nm, 5 μm); column temperature: 25 °C; mobile phase: 0.1% KH_2_PO_4_ solution with pH 5.5; flow rate: 1.0 mL/min; injection volume: 20μL; and measurement wavelength: 265 nm. The encapsulation ratio (EE) and the drug-loading efficiency (LE) were obtained by the following formula:

$$\begin{aligned} {\text{EE}}\left( \% \right) & = \frac{{M_{1} - C_{1} \times L_{1} }}{{M_{1} }} \times 100\% \\ {\text{LE}}\left( \% \right) & = \frac{{M_{1} - C_{1} \times L_{1} }}{{20C_{0} + M_{1} - C_{1} \times L_{1} }} \times 100\% \\ \end{aligned}$$

The total dose of 5-FU was recorded as M_1_, the free 5-FU concentration and volume were recorded as C_1_ and L_1_, and the carrier concentration was recorded as C_0_.

### Characterization

To characterize the prepared nanocomposite, Fourier transform infrared (FT-IR) spectra were scanned from 4000 to 400 cm^−1^ on the IRAffinity-1 spectrometer (Shimadzu, Japan) to confirm the interactions. UV–vis absorption spectra were recorded on a UV-3600 Scanning Spectrophotometer (Shimadzu, Japan). The samples were dissolved in distilled water and the particle sizes, zeta potentials and PDI values were obtained on a Nano-ZS 90 Nano instrument (Malvern, UK). The morphologies of the samples were analyzed using a JEM-2100 transmission electron microscopy (TEM) (JEOL, Japan). Thermogravimetric analyses (TGA) were carried out using a thermo gravimetric analyzer (NETZSCH, Germany) at a heating rate of 10 °C/min from 0 to 800 °C under a nitrogen atmosphere. XRD measurement of samples was carried out by X-ray diffractometer (Bruker, Germany) with a copper CuKα radiation (*λ* = 1.5406 Å) in a wide-angle with 2θ angle. XPS measurements were performed using an Omicron ESCA Probe with a monochromated Al Karadiation (Thermo, America).

### In Vitro Drug Release

In vitro drug release was carried out at pH 1.2 (simulated gastric environments), pH 6.5 (simulated liver cancer cell environment) and pH 7.4 (simulate physiological environment) at 37 °C (simulated body temperature), respectively. In brief, 15 mg of 5-FU-Tau-GO was placed into a dialysis membrane, immersed in 50 mL of buffer solutions containing 0.1% SDS-Na with pH of 1.2, 6.5 and 7.4. All samples were placed in a continuous shaking water bath at a speed of 100 rpm and at a temperature of 37 °C. At predetermined time points (0 min, 5 min, 10 min, 20 min, 30 min, 1 h, 1.5 h, 2 h, 2.5 h, 3 h, 4 h, 8 h, 12 h, 24 h, 48 h and 72 h), 1 mL of each sample was taken and replaced with 1 mL fresh buffer solution containing 0.1% SDS-Na to maintain the same volume of release medium. The released medium was centrifuged and analyzed using 1200 HPLC.

### In Vitro Cytotoxicity Studies

#### MTT Assay

The MTT assay was used to evaluate the cytotoxicity of 5-FU, Tau-GO, and 5-FU-Tau-GO. Briefly, the human hepatoma HepG2 cells were cultured in DEME medium, supplemented with 10% FBS and 1% antibiotics (penicillin–streptomycin solution) at 37 °C in a humidified atmosphere of 5% CO_2_. The cells were seeded into a 96-well plate at a density of 5 × 10^3^ cells per well containing 100 μL of DEME medium, supplemented with FBS and antibiotics. The plate was placed for 24 h into a 37 °C humidified chamber containing 5% CO_2_. After that, the growth medium was removed and refilled with 100 μL fresh medium that contained various concentrations (5, 10, 20, 40, 60, 80 and 100 μg/ml) of 5-FU, Tau-GO, 5-FU-Tau-GO, respectively. After a 24 h incubation, the cells were treated with 20 μL MTT solution and further incubated for 4 h. Next, the medium was aspirated, and the formazan crystals were dissolved in 150 μL DMSO. Finally, the well plates were shaken at 37 °C for 15 min in a constant temperature oscillator. The optical density (OD) of each sample was measured at 570 nm using a microplate reader. The experiments were accomplished in triplicate. The cell inhibition rate was calculated from the results using the following formula:

$${\text{Cell}}\;{\text{inhibition}}\;{\text{rate}} = \frac{{{\text{OD}}_{{{\text{control}}}} - {\text{OD}}_{{{\text{treated}}}} }}{{{\text{OD}}_{{{\text{control}}}} }} \times 100\%$$

where OD_control_ is the absorbance obtained by untreated control cells, OD_treated_ is the absorbance obtained by treated cells.

#### AO/EB Staining Assay

AO/EB double staining was used to evaluate the morphological changes of the cells that were treated with 5-FU, Tau-GO, and 5-FU-Tau-GO. Briefly, HepG2 cells in a logarithmic growth phase were seeded in a 6-well plate at a density of 10,000 cells per well and cultured in an incubator at 37 °C and under a 5% CO_2_ atmosphere. After 24 h, the cells were treated with a fixed concentration of 5-FU, Tau-GO, or 5-FU-Tau-GO, and further incubated for 24 h. The medium in each well was removed and the cells were washed twice with PBS. Next, 1 mL PBS supplemented with 40 μL fluorescent dyes (1 mg/mL AO and 1 mg/mL EB were mixed at a ratio of 1:1) were added to each well and incubated for 10 min without light. The stained cells were observed under a fluorescence microscope and images were randomly taken.

### Pharmacokinetic Study

All animal experiments were conducted in accordance with the policies and principles formulated by the Animal Protection and Ethics Committee. A total of 24 male SD rats, with body weights of 230-270 g, were fasted for 12 h and randomly divided into 4 groups prior to drug administration. Groups A and B were intravenously injected with 5-FU and 5-FU-Tau-GO solutions, and groups C and D were given oral 5-FU and 5-FU-Tau-GO solutions, respectively. All groups received a dose of 20 mg/kg. After drug administration, blood samples (about 0.5 mL) were collected into anticoagulation tubes at given time points (15 min, 1 h, 2 h, 4 h, 6 h, 8 h, 12 h, 16 h, 24 h, and 48 h). The plasma samples were separated by centrifugation at 7500 rpm and at 4 °C for 10 min. Then, 200 μL plasma and 50 mg (NH_4_)_2_SO_4_ were combined in a tube (10 μL of 40 μg/mL 5-BrU internal standard solution, blow dried with nitrogen in a 40 °C water bath, vortexed for 5 min and centrifuged at 10800 rpm for 3 min. Next, the tube was added with 900 μL ethyl acetate/isopropanol solution (85:15, v/v), vortexed for 3 min and centrifuged at 10800 rpm for 15 min. The supernatant was removed and dried with nitrogen, 100 μL of mobile phase was added and the tube was vortexed for 1 min. Finally, the resulting solution collected from the supernatant was measured by 1200 HPLC.

### Molecular Dynamics’ Simulation

Molecular dynamics simulation is mainly used to analyze the interaction force between the drug and the carrier and the diffusion behavior of the drug. The chemical structure of the 5-FU was constructed using Chemdraw of chem office 2014, and the structures of the Tau-GO and GO were conducted in the Polymer and Molecule Builders module using the Materials Studio molecular simulation software (version 7.0, Accelrys Inc., USA). All built compounds were geometrically optimized under the COMPASS II force field, and the conformation with the lowest energy was selected as the stable conformation. A 10 ps, NVT equilibration was conducted for each system. The simulation was performed with 100 ps MD to obtain a balanced structure at 298 K and 101.325 kPa with a step size of 1 fs. Finally, the mean square displacement (MSD) and cohesive energy density (CED) were obtained for each system, and the diffusion coefficient (D) was given by the following formula:

$$D = \frac{1}{2d}\mathop {\lim }\limits_{\tau \to \infty } \frac{{\text{d}}}{{{\text{d}}\tau }}\left[ {\left. {r\overrightarrow {\left( t \right)} - r\overrightarrow {\left( 0 \right)} } \right]} \right.^{2}$$

where the *d* is the dimension of system, $r\overrightarrow {\left( t \right)}$ and $r\overrightarrow {\left( 0 \right)}$ are the position vector of drug molecule at time *t* and 0, respectively, $\left[ {\left. {r\overrightarrow {\left( t \right)} - r\overrightarrow {\left( 0 \right)} } \right]} \right.^{2}$ stands for MSD.

## Results and Discussion

### Characterization

**Fig. 1:**
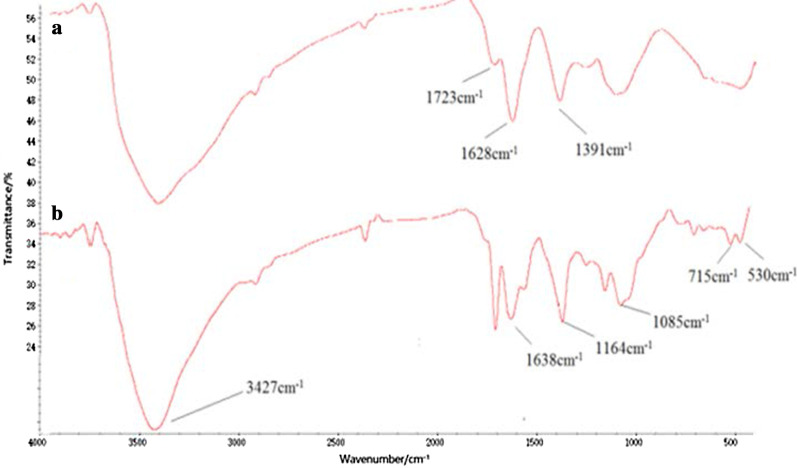
FT-IR spectra of GO (**a**) and Tau-GO (**b**)

**Fig. 2:**
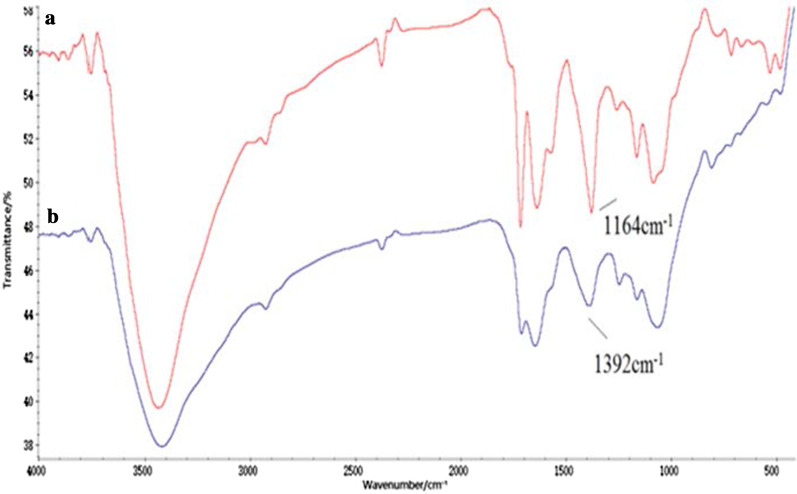
FT-IR spectra of Tau-GO (**a**) and 5-FU-Tau-GO (**b**)

The Tau-GO conjugate was fabricated by an amide bond of GO and Tau. The successful synthesis of the nanocomposite was validated by FT-IR spectra (Fig. 1). The presence of the oxygen functionalities on GO was confirmed by the absorbance peaks at –OH (~ 3405 cm^−1^), C=O (1723 cm^−1^), C=C (1628 cm^−1^) and C–OH (1391 cm^−1^). These results proved the GO successful preparation (Fig. 1a) [25]. In addition to some GO characteristic peaks, the new peaks at 1638 cm^−1^ and 3427 cm^−1^ corresponded to the amide group, and the 1164 cm^−1^ and 1085 cm^−1^ corresponded to –SO. The Tau-GO spectrum clearly indicated that Tau has been functionalized on the GO surface (Fig. 1b). In Fig. 2b, the characteristic peak of –SO_3_ at 1164 cm^−1^ was swamped by the action of hydrogen bonding and was not visible in the FT-IR spectra. Therefore, 5-FU was successfully loaded onto the Tau-GO.

**Fig. 3:**
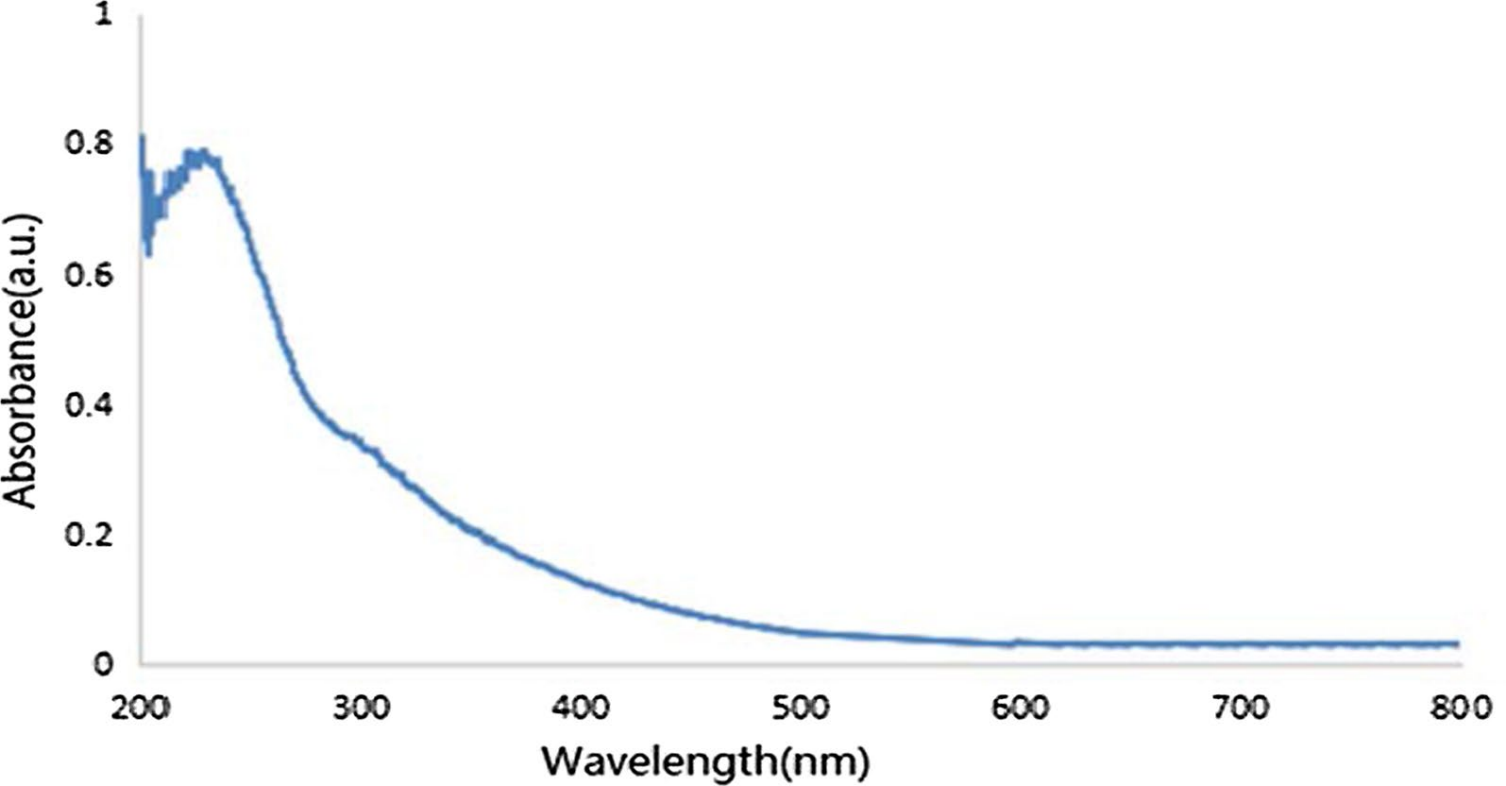
UV spectra of GO

The GO UV–Vis absorption spectra are shown in Fig. 3. The obvious absorption peak at 234 nm was attributed to the π–π* transition of the graphene C=C bonds. In addition, the 300 nm shoulder peak was ascribed to the n–π* transition of the graphene oxide C=O bonds on the carboxyl or the carbonyl group. The two characteristic absorption peaks were proved to be successful preparations of GO.

**Fig. 4:**
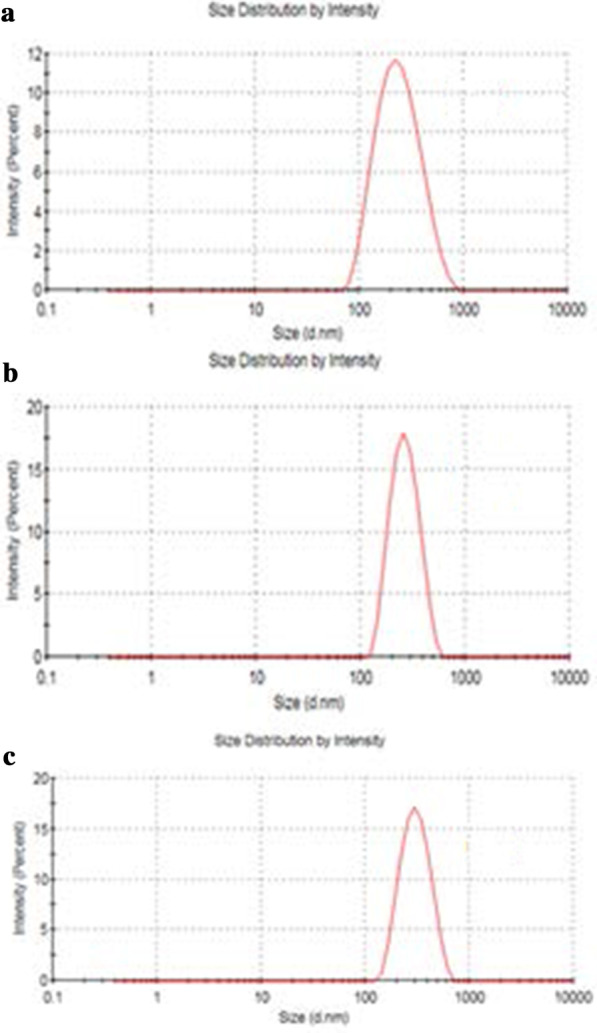
Particle size of GO (**a**), Tau-GO (**b**), and 5-FU-Tau-GO (**c**)

**Fig. 5:**
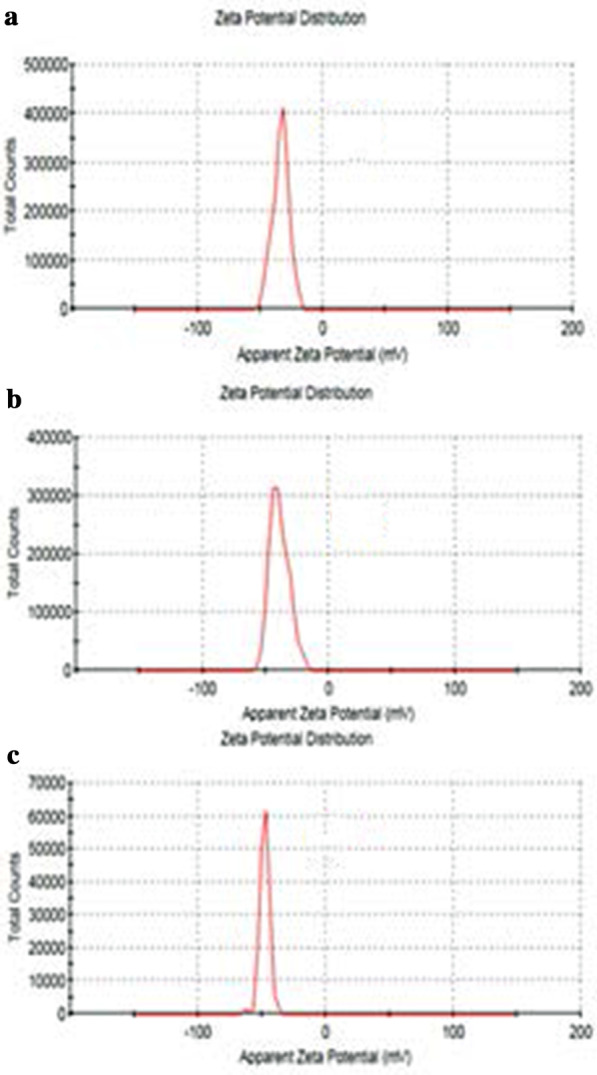
Zeta potentials of GO (**a**), Tau-GO (**b**), and 5-FU-Tau-GO (**c**)

The size of GO, Tau-GO and 5-FU-Tau-GO are shown in Fig. 4. The zeta potential of GO, Tau-GO and 5-FU-Tau-GO are shown in Fig. 5. The size of the GO sheets was approximately 221 nm and the PDI value was approximately 0.188, indicating that the prepared GO has a uniform distribution and good stability. The zeta potential was approximately − 33.3 mV, indicating that the negative charge is mainly due to the presence of many oxygen-containing groups on the surface. When the GO was modified with Tau via amide bond, the amino groups replaced some of the carboxyl groups and the –SO_3_ had a stronger ionization ability in solution, the zeta potential decreased and became − 38.8 mV. The size and PDI values of Tau-GO were approximately 242 nm and 0.190. Next, 5-FU was loaded to Tau-GO by a non-covalent bonding. The zeta potential was approximately − 26.7 mV and the absolute value was greater than 20 mV. Moreover, the size and PDI values of 5-FU-Tau-GO were approximately 264 nm and 0.182, this indicates that the electrostatic repulsion between particles is large, which is not easy to cause aggregation or precipitation, and that Tau has good water solubility, GO also has good water solubility because its surface is modified by oxygen-containing functional groups. The use of Tau-GO carrier to load 5-FU significantly improves the water solubility of 5-FU, so that 5-FU-Tau-GO can be stably dispersed in aqueous solution.

**Fig. 6:**
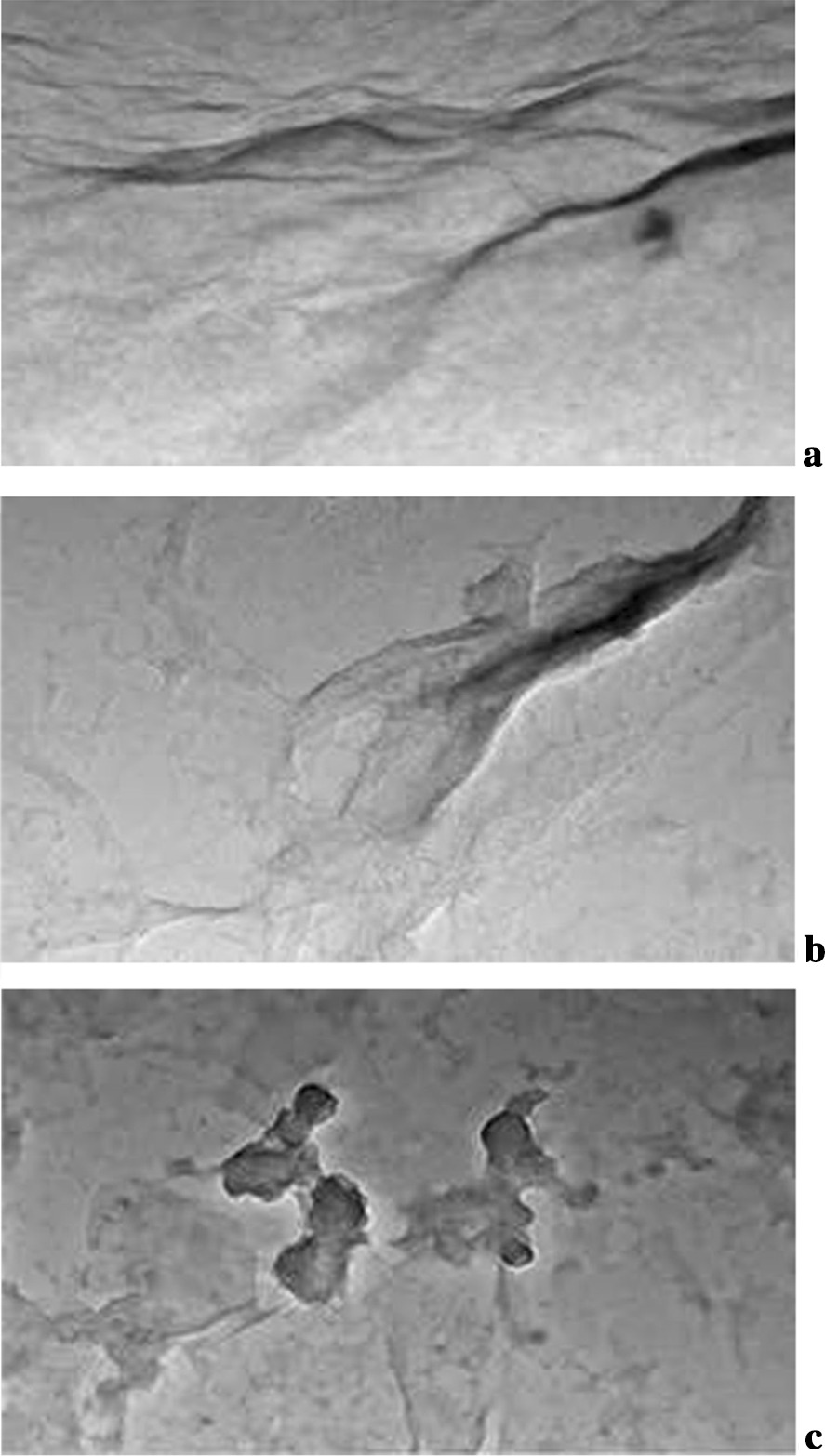
TEM of GO (**a**), Tau-GO (**b**) and 5-FU-Tau-GO (**c**)

The morphologies of GO, Tau-GO and 5-FU-Tau-GO were characterized by TEM (Fig. 6). The GO is a flat structure with wrinkles on the surface, illustrating that it is a planar two-dimensional structure (Fig. 6a). Compared with GO, the size of Tau-GO was slightly increased, but still showed a lamellar structure (Fig. 6b). The 5-FU-Tau-GO image showed that the materials did not aggregate or change with the initial lamellar structure of GO remaining intact (Fig. 6c). Consequently, Tau-GO and 5-FU-Tau-GO had a good stability.

**Fig. 7:**
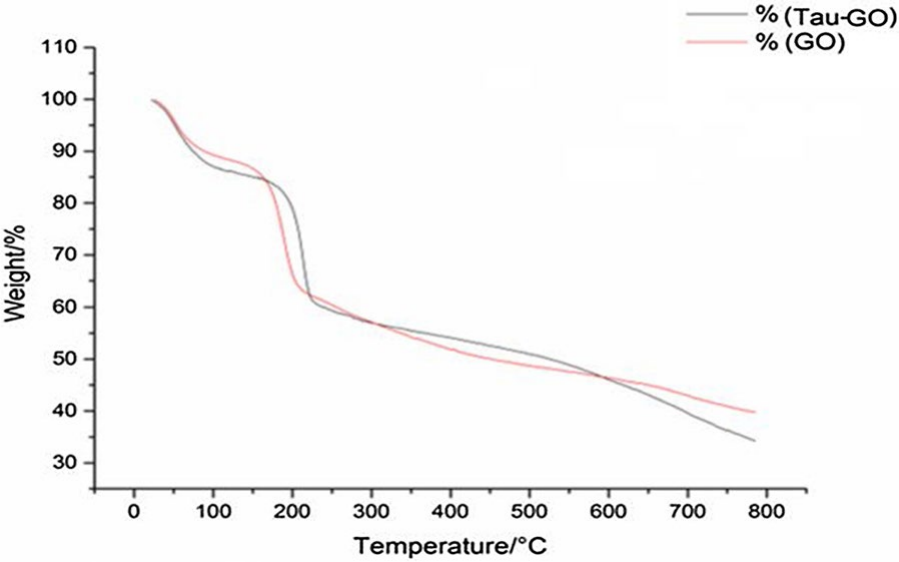
TAG of GO and Tau-GO

Thermogravimetric analysis was used to quantify the compositions of the composites. The TGA curves of GO and Tau-GO are shown in Fig. 7. GO and Tau-GO have remnant masses of 39.73% and 34.22% in a nitrogen atmosphere of 800 °C. Therefore, when comparing the weight change values, the content of Tau in Tau-GO was determined to be approximately 13%.

**Fig. 8:**
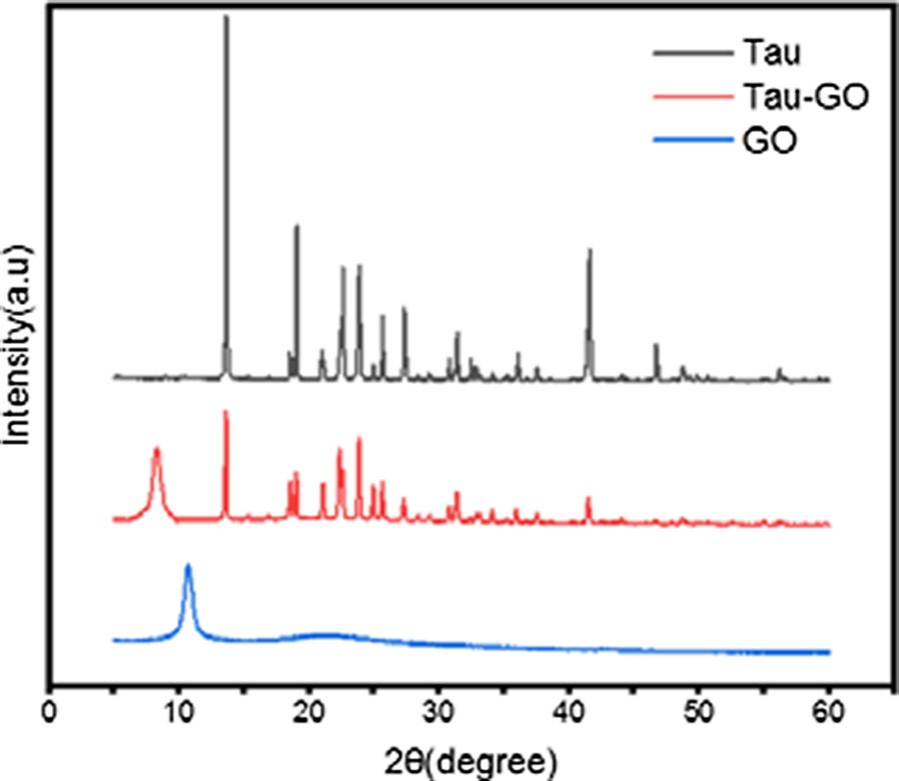
XRD patterns of Tau, Tau-GO and GO

The XRD patterns of Tau, GO and Tau-GO are shown in Fig. 8. The characteristic peak of GO can be observed at 10.7° of the 2θ value, which confirms the formation of GO with complete oxidization for strong chemical oxidation and exfoliation process. After functionalized with Tau on GO surface, the diffraction pattern of peak slightly decreased at 2θ value 8.2. This means that GO has been successfully functionalized by Tau.

**Fig. 9:**
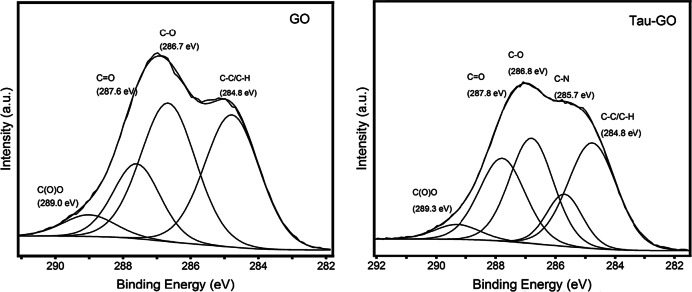
C1s XPS patterns of GO and Tau-GO

The C1s XPS spectra of GO and Tau-GO are showed in Fig. 9. The C1s XPS spectra of GO indicate that there is a considerable degree of oxidation, with the presence of four carbon atoms corresponding to different functional groups. The C1s XPS spectra of Tau-GO are also exhibits these same carbon atoms. In addition, the appearance of C–N bond related component peaks were attributed to the amino groups, amide groups. These results also indicate that GO is successfully functionalized by Tau.

### Drug Loading and Release Behaviors

5-FU was adsorbed onto Tau-GO nanocarrier through non-covalent interactions. The 5-FU calibration curve was *y* = 62.135*x* + 21.873 (*r* = 0.9999), and the range was from 6.5 ~ 250 µg/mL. The encapsulation ratio (EE) and drug-loading efficiency (LE) were determined by unbound drug concentrations to evaluate the drug loading performance. The results showed that EE increased with the increase in drug concentration, and that the highest value of EE was 83.2%. According to the formula, the LE was 33.7%, that is 508.52 μg of 5-FU can be adsorbed on 1 mg of Tau-GO. Therefore, Tau-GO is a promising drug carrier that can achieve a large drugs’ load. The possible mechanisms of the high loading capacity of 5-FU onto the Tau-GO can be summarized by the following explanations: firstly, Tau is used to functionalize GO and introduce active functional groups (–SO_3_). –SO_3_ has a strong ionization ability in the solution, which reduces the agglomeration between GO and facilitates the loading of 5-FU into Tau-GO. Secondly, the Zeta potential of 5-FU-Tau-GO is 12.1 mV different from that of Tau-GO, which indicates that 5-FU is loaded onto the surface of Tau-GO, and the electrostatic interaction plays an important role in the loading of 5-FU. Lastly, there are many forms of hydrogen bonds between 5-FU and Tau-GO carrier, including –COOH in Tau-GO and –NH– in 5-FU, –COOH in Tau-GO and-in 5-FU C=O, –OH in Tau-GO and –NH– in 5-FU, –OH in Tau-GO and –C=O in 5-FU, –COOH and 5-FU in Tau-GO –F in Tau-GO, –COOH in Tau-GO and –F in 5-FU, these hydrogen bonds make 5-FU-Tau-GO stable in solution.

**Fig. 10:**
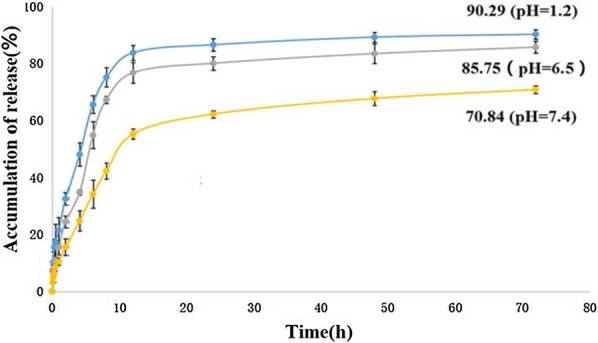
In vitro release curves of 5-FU-Tau-GO in phosphate saline at 37 °C

The in vitro cumulative release of 5-FU from the 5-FU-Tau-GO, at the temperature of 37 °C in pH 1.2, 6.5 and 7.4 PBS solution (simulate gastric environment, liver cancer cell environment and physiological environment, respectively) is shown in Fig. 10. It is found that the 5-FU released behavior was affected by the pH value of the environment. In a pH 7.4 buffer, the drug release was slow and continuous, and the total released amount of drug was approximately 70.84% after 72 h. In contrast, the released amount of drug at pH 7.4 was significantly lower than that at pH 1.2 and pH 6.5 at the same time point. The total drug loading that was released from the 5-FU-Tau-GO could be achieved at approximatively 90.29% and 85.75% and at pH 1.2 and pH 6.5, respectively. It can be attributed to the π-π interactions and hydrogen bonds between 5-FU and Tau-GO. The lower the pH value, the higher the degree of protonation of the hydrogen bond. Therefore, the hydrogen bond strength was controlled by pH value, which led to the release of 5-FU. This pH-sensitive drug delivery system plays an important role in anti-tumor drugs and can achieve the drugs’ release into the tumor cell.

### In Vitro Cytotoxicity Studies

**Fig. 11:**
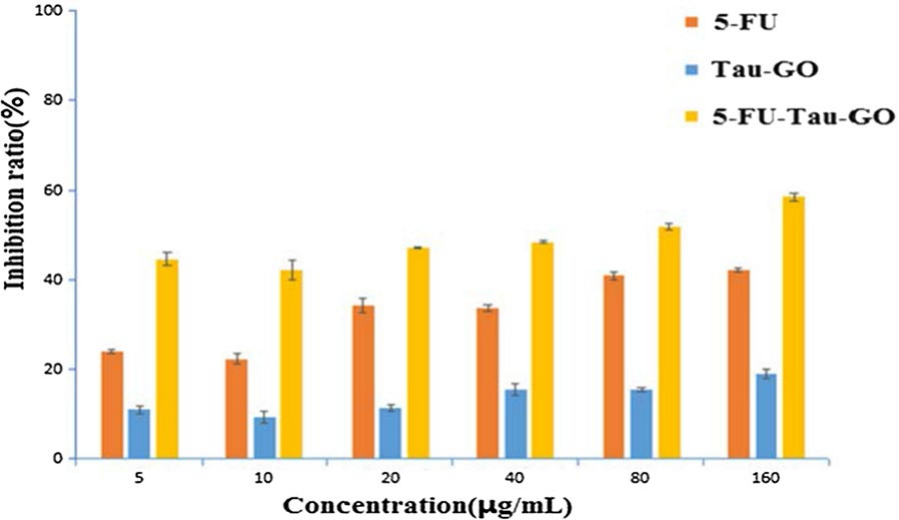
The viability of different concentrations of 5-FU, Tau-GO, and 5-FU-Tau-GO

To evaluate the potential toxicity and the tumor therapy efficacy of nanocarrier, in vitro cell viability was carried out in HepG2 cells using MTT assays (Fig. 11). The Tau-GO did not show significant cytotoxicity at different concentrations. After 5-FU loading, the 5-FU-Tau-GO showed an obvious inhibition effect, and in dose-dependent manner, therefore, the nanocarrier had the ability to deliver antitumor drugs. The IC_50_ value of 5-FU-Tau-GO was 65.2 ± 0.7 μg/mL, which was more toxic than the free 5-FU (196 ± 8.73 μg/mL). This may be due to taurine’s capacity to induce apoptosis in tumor cells, thereby indirectly enhancing the inhibitory effect of 5-FU on cells. Furthermore, it could be seen from the in vitro release experiment that 5-FU loaded on the Tau-GO could be released gradually in the cells. Therefore, the effective time of 5-FU-Tau-GO on cells was longer than that of free 5-FU, and thus produced a better inhibition.

**Fig. 12:**
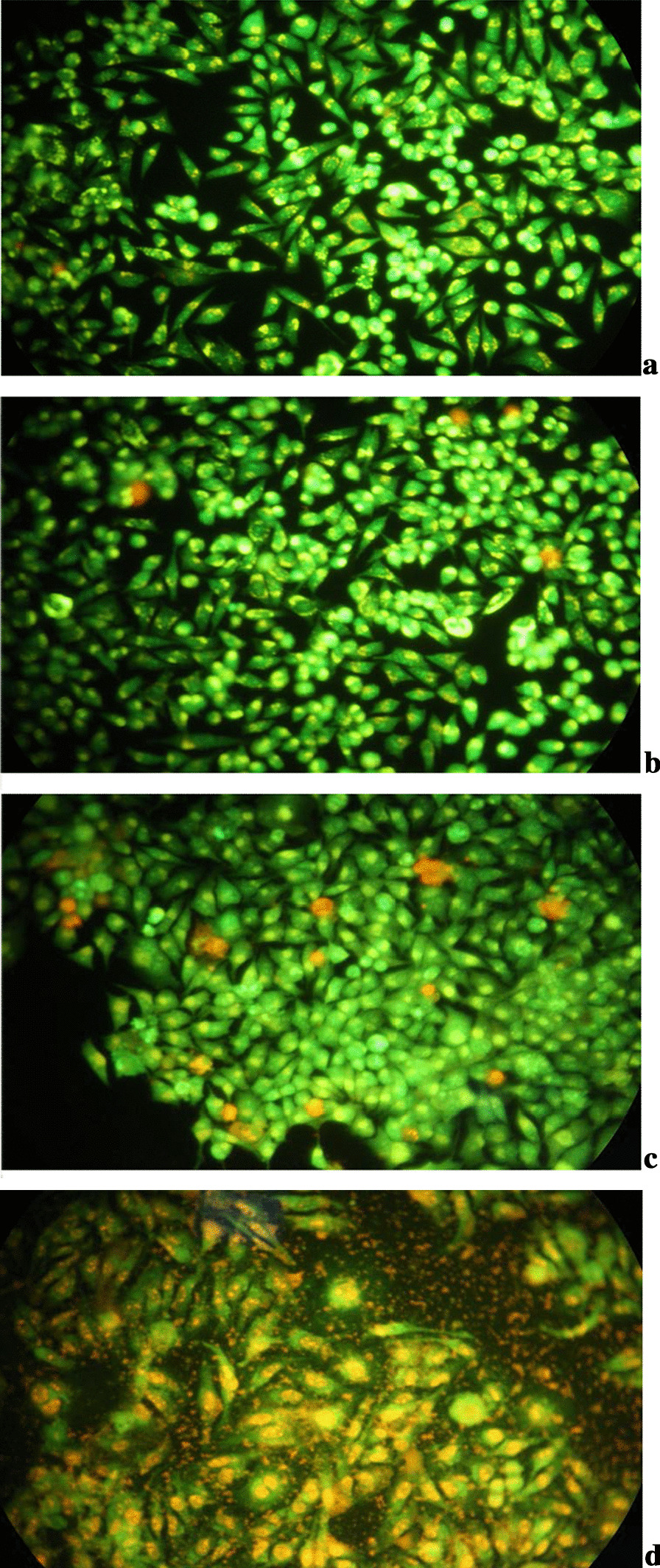
The AO/EB of control (**a**), Tau-GO (**b**), 5-FU (**c**), and 5-FU-Tau-GO (**d**)

AO fluorescent agent could emit green fluorescence when it passed through intact cell membranes and stained nuclei, while EB only marked the nucleus of damaged cells that emitted a red/orange fluorescence. The cells with early and late apoptosis presented greenish yellow or orange nuclei with the AO/EB stain, respectively. Therefore, AO/EB staining was performed to investigate whether the cells death was associated with apoptosis using characteristics of cell morphological changes. The results obtained from the AO/EB staining are presented in Fig. 12. Control cells were in spindle shape and with green nuclei. In the cell group that was cultured with Tau-GO alone, small parts of the nuclei were invaginated and with dark green or orange-red staining. Significant orange or red apoptotic cells with chromatin fragments and apoptotic bodies were observed in the 5-FU alone group. Compared with 5-FU, 5-FU-Tau-GO caused more damage to HepG2 cell morphology, which not only broke the cells, but also caused a large amount of apoptosis in cancer cells. As can be seen from the pictures, almost all the cells that were treated with 5-FU-Tau-GO, had morphological changes, a large number of cell debris and apoptotic bodies, indicating that the 5-FU-Tau-GO nano drug delivery system had a good killing effect on HepG2 cells.

### Pharmacokinetic Studies

**Fig. 13:**
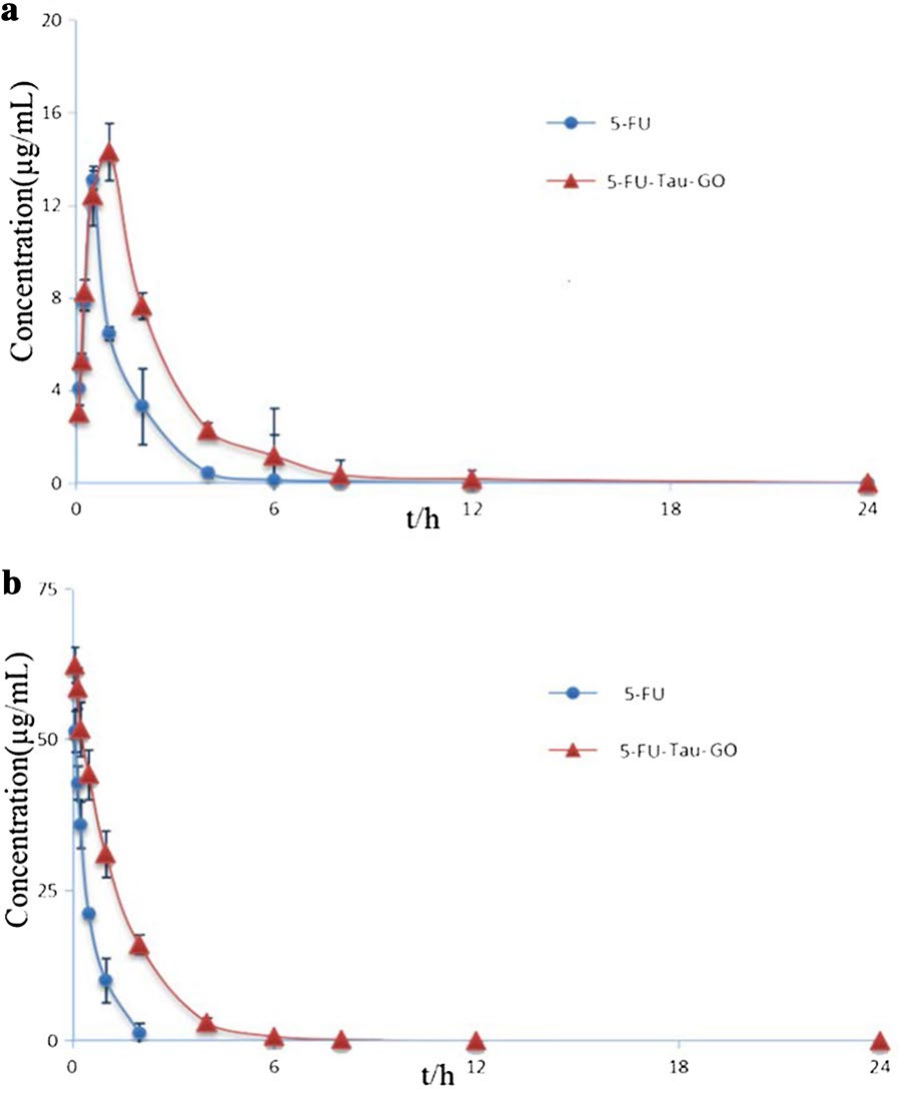
In vivo pharmacokinetic standard curves of 5-FU and 5-FU-Tau-GO through oral administration (**a**). In vivo pharmacokinetic standard curve of 5-FU and 5-FU-Tau-GO through intravenous injection (**b**)

The pharmacokinetic studies of 5-FU and 5-FU-Tau-GO were performed in SD rats. The profiles of 5-FU concentration in plasma vs. time, following oral administration, are presented in Fig. 13a. We found that the tendency of the two curves was similar, but the 5-FU plasma concentration from the 5-FU-Tau-GO nanocarrier was higher than that from the 5-FU alone and this was observed at each measured time point. Figure 13b shows the 5-FU in vivo release profiles via tail vein. The 5-FU-Tau-GO could achieve sustained drug release over 24 h, and the drug concentration gradually decreased in the first few hours, indicating that 5-FU was slowly released.

The two-compartment model was used to analyze the pharmacokinetic parameters of oral or intravenous administration in rats. The pharmacokinetic parameters are presented in Table 1. Compared with the 5-FU, the 5-FU-Tau-GO showed higher T_1/2β_ that were 2.3 times by oral administration, and 3.0 times by intravenous injection, respectively. Moreover, the area under the concentration time curve (AUC_0−t_) of 5-FU-Tau-GO nanocomplexes was roughy 2.1-fold higher through the oral administration, and 2.8-fold higher through intravenous injection when compared to that of the 5-FU solution, respectively. Therefore, we concluded that 5-FU-Tau-GO could significantly extend 5-FU retention time in vivo and improve bioavailability. In addition, the T_1/2β_ of the 5-FU-Tau-GO nanocomplexes that were orally administered (1.67 ± 1.15 h), was longer than that of the intravenous injection (1.33 ± 0.64 h); however, the AUC_0−t_ of oral administration (36.02 ± 1.83 mg/L*h) was lower than that of intravenous injection (96.50 ± 8.70 mg/L*h). These results might be due to two aspects: on the one hand, when administered by intravenous injection, the drug directly enters the blood system for circulation and without passing through the gastrointestinal barrier for redistribution; on the other hand, because 5-FU easily causes a certain damage to the gastrointestinal system, it may also affect the effective use of drugs in the body.

**Table 1:** The basic pharmacokinetic parameters of 5-FU and 5-FU-Tau-GO through oral administration and intravenous injection

Parameter	Unit	Oral administration (5-FU)	Oral administration (5-FU-Tau-GO)	Intravenous injection (5-FU)	Intravenous injection (5-FU-Tau-GO)
T_1/2α_	h	0.31 ± 0.5	0.41 ± 0.22	0.22 ± 0.12	0.75 ± 0.42
T_1/2β_	h	0.73 ± 0.46	1.67 ± 1.15	0.44 ± 0.04	1.33 ± 0.64
V_1/F_	L/kg	1.05 ± 0.04	0.62 ± 0.19	0.38 ± 0.06	0.38 ± 0.01
CL/F	L/h/kg	1.45 ± 0.09	0.69 ± 0.04	0.62 ± 0.02	0.25 ± 0.02
AUC_(0−t)_	mg/L*h	17.12 ± 1.11	36.02 ± 1.83	34.62 ± 1.00	96.50 ± 8.70
AUC_(0−∞)_	mg/L*h	17.33 ± 1.17	36.30 ± 1.94	40.30 ± 1.04	102.34 ± 8.71
K_10_	1/h	1.38 ± 0.11	1.20 ± 0.36	1.66 ± 0.34	0.66 ± 0.07
K_12_	1/h	1.68 ± 1.11	0.25 ± 0.12	1.50 ± 1.92	0.23 ± 0.26
K_21_	1/h	1.88 ± 0.09	1.23 ± 0.80	3.25 ± 1.79	1.38 ± 1.64

### MD Simulations

**Fig. 14:**
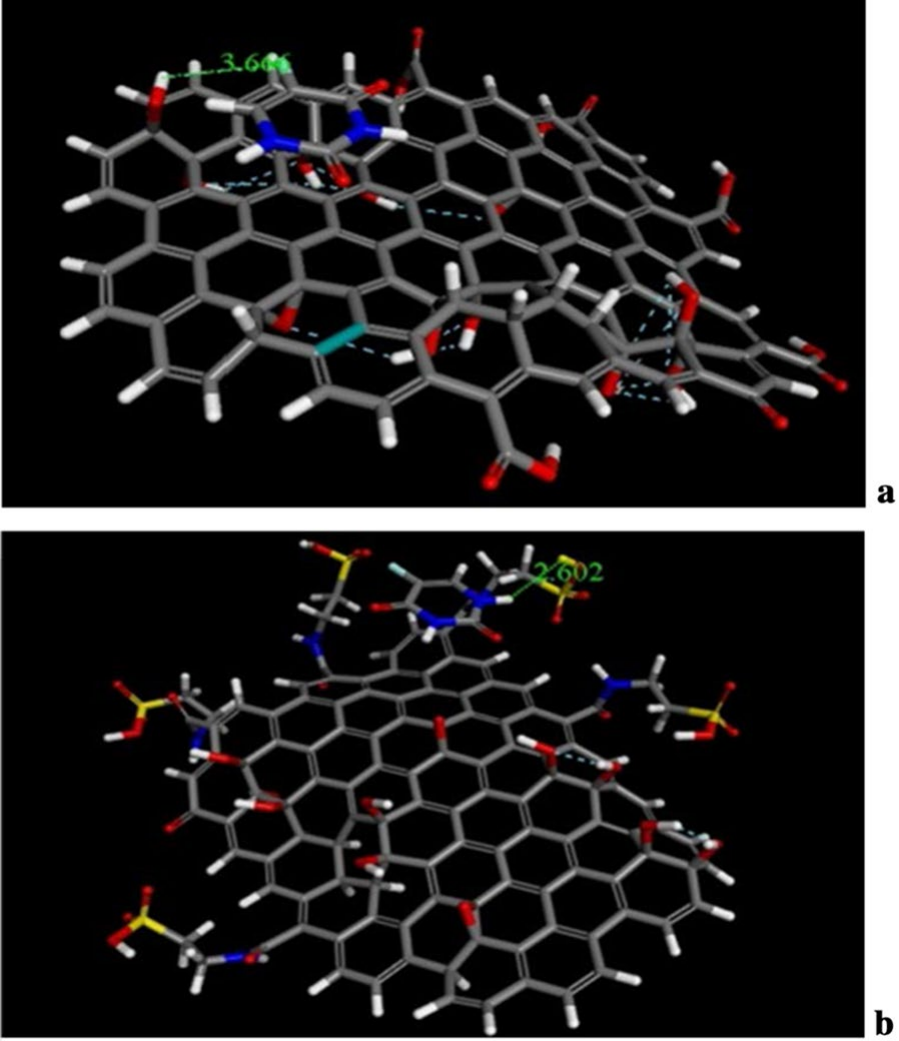
The Molecular docking of GO sheets with 5-FU (**a**). The molecular docking of Tau-GO with 5-FU (**b**)

The docking and molecular dynamics of unmodified GO, Tau-GO and 5-FU were simulated by molecular docking and molecular dynamics simulation. The molecular docking results of GO, Tau-GO and 5-FU are shown in Fig. 14, where it can be seen that the bond lengths of 5-FU and GO and Tau-GO are 3.66 Å and 2. 602 Å, respectively. Moreover, from the calculation results, the binding energies of 5-FU to GO and Tau-GO were 47.69 kcal/mol and 25.04 kcal/mol, respectively. These indicated that the binding force of Tau-GO and 5-FU was stronger than that of GO and 5-FU. This is due to Tau polar atoms, such as S and N, forming a stronger non-covalent bond with 5-FU, that makes the force between Tau-GO and 5-FU stronger.

**Fig. 15:**
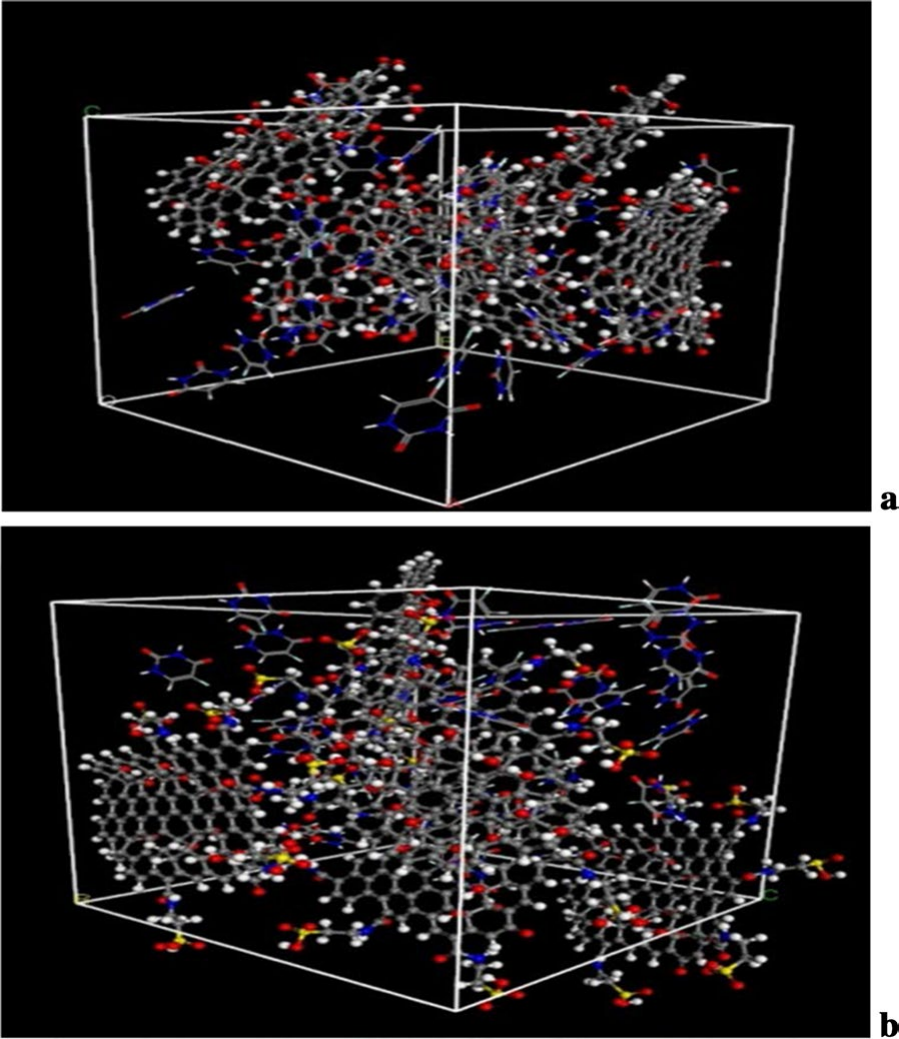
Snapshots of the GO and 5-FU at the end of the MD (**a**). Snapshots of the Tau-GO and 5-FU at the end of the MD (**b**)

**Fig. 16:**
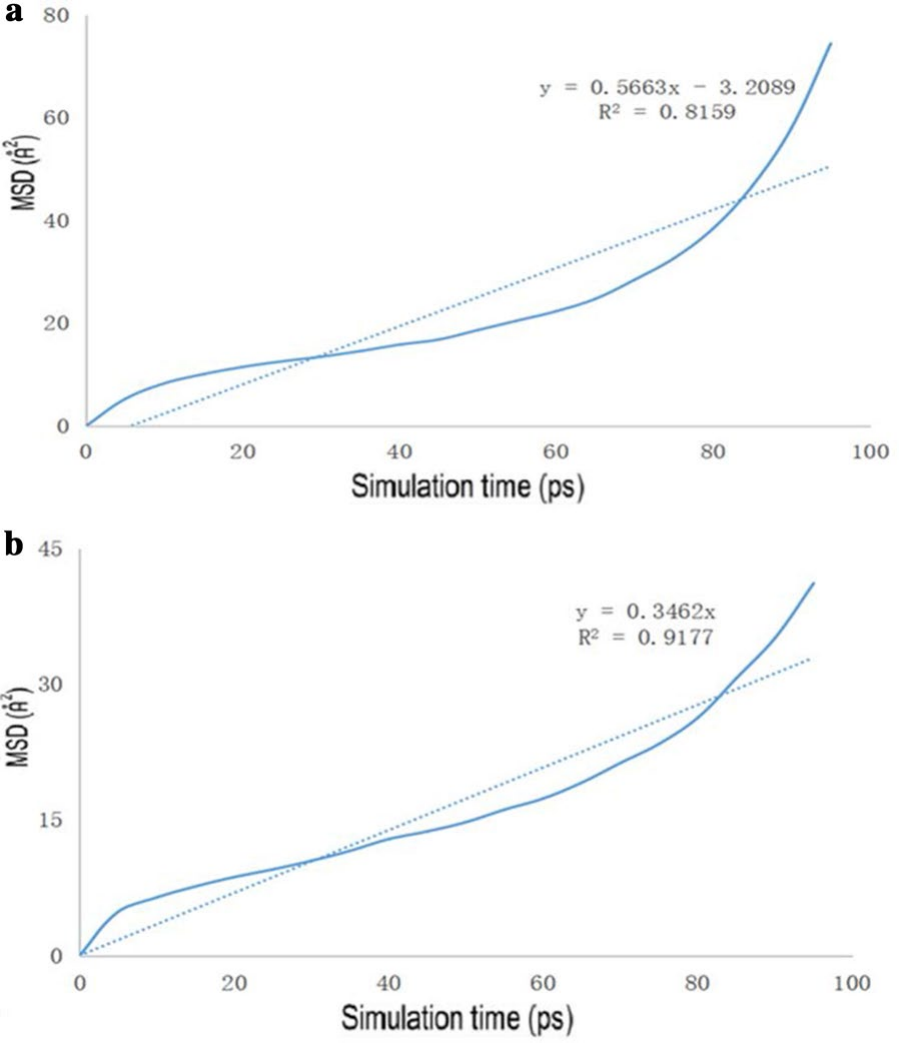
The drug MSD profiles of the GO and 5-FU (**a**), Tau-GO and 5-FU (**b**)

The diagrams of the molecular dynamics simulation of GO, Tau-GO and 5-FU were shown in Fig. 15. According to the calculation results, the CED of 5-FU-GO and 5-FU-Tau-GO were 2.67*10^8^ and 2.83*10^8^, respectively. These results showed a stronger interaction between the drug and the Tau-GO. The graphs between MSD and time were plotted (Fig. 16) to obtain the diffusion coefficient via MSD. The drug diffusion coefficients were obtained by the slope divided by 6 as follows: 0.094m^2^/s and 0.058 m^2^/s. These show that the force between Tau-GO and 5-FU is stronger, which is consistent with the results of the molecular docking. Therefore, the functionalized GO makes the entire carrier more abundant in atoms and groups; Therefore, making the non-covalent bond with 5-FU stronger, and the entire system more stable.

## Conclusions

In summary, we successfully prepared a Tau-GO nanocomposite through a simple chemical method. GO functionalization with Tau has a good stability and improves its biocompatibility. The unique structure and brilliant properties of Tau-GO nanocarriers offer great opportunities for the loading and delivery of 5-FU. The 5-FU-Tau-GO has a potential anti-tumor ability and an excellent circulation time of drugs. Therefore, we believe that the modification of GO by the carrier Tau for 5-FU loading, is an effective and applicable tool for constructing a 5-FU-Tau-GO nano drug delivery system for the delivery of anticancer drugs and anti-tumor therapy.

## Data Availability

The datasets used and/or analyzed during the current study are available from the corresponding author on reasonable request.

## Abbreviations

GO: Graphene oxide
Tau: Taurine
5-FU: 5-Fluorouracil
5-FU-Tau-GO: Taurine functionalized graphene oxide loading 5-fluorouracil
EE: Encapsulation ratio
LE: Drug-loading efficiency
FT-IR: Fourier transform infrared
TEM: Transmission electron microscopy
TGA: Thermogravimetric analyses
MSD: Mean square displacement
CED: Cohesive energy density
